# Highly Reduced Complementary Genomes of Dual Bacterial Symbionts in the Mulberry Psyllid *Anomoneura mori*

**DOI:** 10.1264/jsme2.ME24041

**Published:** 2024-09-06

**Authors:** Yuka Yasuda, Hiromitsu Inoue, Yuu Hirose, Atsushi Nakabachi

**Affiliations:** 1 Department of Applied Chemistry and Life Science, Toyohashi University of Technology, 1–1 Hibarigaoka, Tempaku, Toyohashi, Aichi 441–8580, Japan; 2 Institute for Plant Protection, National Agriculture and Food Research Organization, Higashihiroshima, Hiroshima 739–2494, Japan; 3 Research Center for Agrotechnology and Biotechnology, Toyohashi University of Technology, 1–1 Hibarigaoka, Tempaku, Toyohashi, Aichi 441–8580, Japan

**Keywords:** bacteriome, genome reduction, metabolic complementarity, convergence, ancient symbionts

## Abstract

The genomes of obligately host-restricted bacteria suffer from accumulating mildly deleterious mutations, resulting in marked size reductions. Psyllids (Hemiptera) are phloem sap-sucking insects with a specialized organ called the bacteriome, which typically harbors two vertically transmitted bacterial symbionts: the primary symbiont “*Candidatus* Carsonella ruddii” (*Gammaproteobacteria*) and a secondary symbiont that is phylogenetically diverse among psyllid lineages. The genomes of several *Carsonella* lineages were revealed to be markedly reduced (158–174‍ ‍kb), AT-rich (14.0–17.9% GC), and structurally conserved with similar gene inventories devoted to synthesizing essential amino acids that are scarce in the phloem sap. However, limited genomic information is currently available on secondary symbionts. Therefore, the present study investigated the genomes of the bacteriome-associated dual symbionts, Secondary_AM (*Gammaproteobacteria*) and *Carsonella*_AM, in the mulberry psyllid *Anomoneura mori* (Psyllidae). The results obtained revealed that the Secondary_AM genome is as small and AT-rich (229,822 bp, 17.3% GC) as those of *Carsonella* lineages, including *Carsonella*_AM (169,120 bp, 16.2% GC), implying that Secondary_AM is an evolutionarily ancient obligate mutualist, as is *Carsonella*. Phylogenomic ana­lyses showed that Secondary_AM is sister to “*Candidatus* Psyllophila symbiotica” of *Cacopsylla* spp. (Psyllidae), the genomes of which were recently reported (221–237‍ ‍kb, 17.3–18.6% GC). The Secondary_AM and *Psyllophila* genomes showed highly conserved synteny, sharing all genes for complementing the incomplete tryptophan biosynthetic pathway of *Carsonella* and those for synthesizing B vitamins. However, sulfur assimilation and carotenoid-synthesizing genes were only retained in Secondary_AM and *Psyllophila*, respectively, indicating ongoing gene silencing. Average nucleotide identity, gene ortholog similarity, genome-wide synteny, and substitution rates suggest that the Secondary_AM/*Psyllophila* genomes are more labile than *Carsonella* genomes.

Animals and microbes have diverse symbiotic relationships, among which the most intimate are bacteriome-associated mutualisms in insects (Moran *et al.*, 2008; McCutcheon *et al.*, 2019; Alarcón *et al.*, 2022). Various insect lineages, particularly those feeding on nutritionally restricted diets, such as plant sap and vertebrate blood, possess the bacteriome, a specialized organ with apparently varied developmental origins (Alarcón *et al.*, 2022), which harbors the ‘primary symbiont,’ providing a nutritional supply to support the survival of holobionts (host-symbiont complexes). Distinct insect lineages harbor phylogenetically diverse primary symbionts, indicating their independent origins from various free-living microbes. They are mostly bacterial and feature organelle-like characteristics, including intracellular localization within the host, complete infection in host populations, host-symbiont cospeciation through strict vertical transmission, and marked genome reductions due to the elevated fixation of deleterious mutations and the resulting loss of non-essential genes (Moran *et al.*, 2008; McCutcheon *et al.*, 2019). Their highly reduced genomes retain many of the genes required to synthesize essential nutrients that fulfill host requirements. However, the contingent acquisition of additional symbionts with a functionally intact genome may further allow the erosion of primary symbiont genomes to a level where the genes essential for the holobiont’s survival are also degraded and complemented by newcomers. In that case, the primary symbionts *per se* may eventually be replaced by the newly acquired symbionts. Apparent snapshots of these evolutionary processes have been observed in several insect lineages (McCutcheon *et al.*, 2009; Vogel and Moran, 2013; Bennett *et al.*, 2014; Monnin *et al.*, 2020; Michalik *et al.*, 2021).

Psyllids (Hemiptera: Sternorrhyncha: Psylloidea) are phloem sap-sucking insects encompassing ~4,000 described species worldwide (Burckhardt *et al.*, 2021). They have a large bilobed bacteriome within the abdominal hemocoel (Profft, 1937; Chang and Musgrave, 1969; Waku and Endo, 1987; Nakabachi *et al.*, 2010; Dan *et al.*, 2017; Nakabachi and Suzaki, 2023), which typically harbors two distinct bacterial symbionts (Subandiyah *et al.*, 2000; Spaulding and von Dohlen, 2001; Hall *et al.*, 2016; Morrow *et al.*, 2017; Nakabachi *et al.*, 2022a; Dittmer *et al.*, 2023; Maruyama *et al.*, 2023). The primary symbiont is “*Candidatus* Carsonella ruddii” (*Gammaproteobacteria*: *Oceanospirillales*, hereafter *Carsonella*) (Thao *et al.*, 2000b; Nakabachi and Moran, 2022), which has been detected in all psyllid species analyzed to date and is, thus, considered to be essential for Psylloidea. Molecular phylogenetic ana­lyses demonstrated cospeciation between *Carsonella* lineages and their host psyllids, resulting from the single acquisition of an ancestor of *Carsonella* by a psyllid common ancestor and its subsequent strict vertical transmission (Thao *et al.*, 2000b; Spaulding and von Dohlen, 2001; Hall *et al.*, 2016; Morrow *et al.*, 2017; Maruyama *et al.*, 2023). *Carsonella* genomes derived from several psyllid lineages were analyzed and revealed to be markedly reduced in size (158–174‍ ‍kb), AT-rich (14.0–17.9% GC), and structurally conserved. They lack numerous genes that are apparently essential for bacterial life but retain genes to synthesize essential amino acids that are deficient in the phloem sap diet (Nakabachi *et al.*, 2006, 2013b, 2020b; Nakabachi, 2008; Sloan and Moran, 2012; Dittmer *et al.*, 2023). Another bacterial lineage housed in the bacteriome is categorized as a ‘secondary symbiont’, which is phylogenetically diverse among psyllid lineages, suggesting its repeated infection and replacement during the evolution of Psylloidea (Thao *et al.*, 2000a; Spaulding and von Dohlen, 2001; Sloan and Moran, 2012; Hall *et al.*, 2016; Morrow *et al.*, 2017; Nakabachi *et al.*, 2020a, 2022a, 2022b). Although secondary symbionts in diverse insect taxa form various host-symbiont relationships across the mutualism-parasitism continuum (Dale and Maudlin, 1999; Nakabachi *et al.*, 2003; Werren *et al.*, 2008; Oliver *et al.*, 2010; Johnson, 2015), those in the psyllid bacteriome appear to be consistently hosted by all individuals within a particular psyllid species, having obligate mutualistic relationships with the host psyllid. Therefore, they are occasionally called ‘co-primary symbionts,’ as in various auchenorrhynchan insects (McCutcheon *et al.*, 2009; Bennett *et al.*, 2014; Michalik *et al.*, 2021). In contrast to *Carsonella*, limited genomic information is currently available on secondary symbionts associated with psyllid bacteriomes. Sloan and Moran exami­ned the whole genome sequences of two distantly related secondary symbionts (both *Gammaproteobacteria*: *Enterobacterales*) derived from *Ctenarytaina eucalypti* (Aphalaridae: Spondyliaspidinae) and *Heteropsylla cubana* (Psyllidae: Ciriacreminae) (Sloan and Moran, 2012). The findings obtained showed that they are larger with less biased nucleotide contents (1,441‍ ‍kb with 43.3% GC in *Ct. eucalypti*; 1,122‍ ‍kb with 28.9% GC in *H. cubana*) than other insect bacteriome-associated symbionts analyzed thus far, including *Carsonella*, suggesting their recently formed relationships with the host psyllid. Both genomes encoded genes to complement the incomplete essential amino acid biosynthetic pathways encoded in the‍ ‍genome of co-residing *Carsonella* (Sloan and Moran, 2012). Subsequent ana­lyses of two lineages of “*Candidatus* Profftella armatura” (*Betaproteobacteria*: *Burkholderiales*, hereafter *Profftella*) from the Asian citrus psyllid *Diaphorina citri* and its relative *Diaphorina* cf. *continua* (both Psyllidae: Diaphorininae) revealed that their genomes are markedly reduced in size and AT-rich (460‍ ‍kb with 24.2% GC in *D. citri*; 470‍ ‍kb with 24.4% GC in *D.* cf. *continua*) (Nakabachi *et al.*, 2013a, 2013b, 2020b), suggesting that *Profftella* is an ancient symbiont. The highly conserved synteny and comparatively low substitution rates of the genomes implied that *Profftella* acquired a relatively stable status, which is similar to that of *Carsonella*. These genomes shared all genes for the biosynthesis of toxins (diaphorin and hemolysin), carotenoids, and B vitamins (riboflavin and biotin), indicating that *Profftella* is a unique versatile symbiont that plays multiple roles, including nutritional supply and defenses against natural enemies. Subsequent studies demonstrated the distinct activities of diaphorin, such as inhibitory effects against divergent organisms (Nakabachi and Fujikami, 2019; Nakabachi and Okamura, 2019; Yamada *et al.*, 2019; Tanabe *et al.*, 2022; Takasu *et al.*, 2023, 2024). These collective benefits to the host may contribute to the stabilization of symbiotic relationships, leading to the organelle-like status of *Profftella*. However, these four lineages represent only a small fraction of the diverse secondary symbionts associated with the psyllid bacteriome; therefore, their evolutionary behaviors and the number of versatile symbionts, such as *Profftella*, in Psylloidea remain unclear.

As the first step to obtaining further insights into the evolution of the dual symbiotic system of the psyllid bacteriome, we analyzed the genomes of the bacteriome-associated secondary symbiont (*Gammaproteobacteria*: *Enterobacterales*, hereafter Secondary_AM) and *Carsonella* (hereafter *Carsonella*_AM) of a sericultural pest, the mulberry psyllid *Anomoneura mori* (Psyllidae: Psyllinae), in which the localization of symbionts has already been reported (Fukatsu and Nikoh, 1998). During the ana­lysis, the genomes of 12 *Carsonella* lineages and 10 line­ages‍ ‍of‍ ‍a‍ ‍secondary symbiont “*Candidatus* Psyllophila symbiotica” (*Gammaproteobacteria*: *Enterobacterales*, hereafter *Psyllophila*) from four *Cacopsylla* spp. (Psyllidae: Psyllinae) were recently published, revealing that the *Psyllophila* genomes are markedly reduced in size (221–237‍ ‍kb) and AT-rich (17.3–18.6% GC), encoding genes complementary to those of *Carsonella* (Dittmer *et al.*, 2023). We compared the genomes of the bacteriome-associated symbionts of *A. mori* with the previously sequenced genomes of psyllid symbionts.

## Materials and Methods

### Insect specimen and DNA preparation

The material of *A. mori* was collected from the mulberry tree *Morus* sp. (Moraceae) in Tsukuba city, Ibaraki Prefecture, Honshu, Japan (36.048N, 140.101E, 23‍ ‍m‍ ‍a.s.l.) on May 26, 2015. DNA was extracted from a pool of the bacteriomes of three adult female *A. mori* using a DNeasy Blood & Tissue Kit (Qiagen) following the manufacturer’s instructions. Whole genome amplification was performed using extracted DNA and a REPLI-g Mini Kit (Qiagen) according to the manufacturer’s instructions.

### Sequencing and assembly

An 800-bp paired-end library and an 8-kbp mate-pair library of *A. mori* bacteriome DNA were prepared using the TruSeq DNA PCR-Free Sample Preparation kit (Illumina) and Nextera Mate Pair Sample Preparation kit (Illumina), respectively. The libraries were sequenced on the MiSeq instrument (Illumina) with the MiSeq Reagent kit v3 (600-cycles; Illumina). Paired reads in which either of the pair showed similarity (e-value scores <1.0E-5) to the previously reported genomic sequences of eight *Carsonella* lineages (NC_018417.1, AP009180.1, CP003541.1, CP003542.1, CP003543.1, CP003545.1, CP003467.1, and CP012411.1) were collected from the sequences obtained by a local alignment search with the Nucleotide Basic Local Alignment Search Tool (BLASTN) program (Camacho *et al.*, 2009) using a custom Perl script. Sequencing errors were corrected using ShortReadManager based on 17-mer frequency (Ohtsubo *et al.*, 2022). Collected and refined reads were assembled using Newbler version 2.9 (Roche). Gap sequences between contigs were assessed *in silico* using GenoFinisher and AceFileViewer (Ohtsubo *et al.*, 2022).

### Annotation and structural ana­lysis of genomes

Initial gene predictions and annotations were conducted using the DNA Data Bank of Japan (DDBJ) Fast Annotation and Submission Tool (DFAST) pipeline version 1.2.0 (Tanizawa *et al.*, 2018) and the National Center for Biotechnology Information (NCBI) Prokaryotic Genome Annotation Pipeline (PGAP) version 2023-05-17.build6771 (Tatusova *et al.*, 2016), followed by manual corrections with the aid of Rfam version 12.2 (Kalvari *et al.*, 2021), the NCBI ORFfinder (Wheeler *et al.*, 2003), BLAST (Camacho *et al.*, 2009), and eggNOG-mapper version 2.1.12 (Cantalapiedra *et al.*, 2021). The functional categories of Cluster of‍ ‍Orthologous Groups (COG) were assigned to predicted genes‍ ‍using the abovementioned version of eggNOG-mapper (Cantalapiedra *et al.*, 2021). Metabolic pathways were analyzed using the Kyoto Encyclopedia of Genes and Genomes (KEGG) (Kanehisa, 2019). Pathway maps were created by examining the presence or absence of genes involved in the biosynthesis of essential amino acids and vitamins. Dinucleotide bias and GC skew were analyzed and circular diagrams were drawn using ArcWithColor version 1.62 (Ohtsubo *et al.*, 2008). The codon adaptation index (CAI) was calculated using the CAIcal server (Puigbo *et al.*, 2008). Pairwise comparisons of genomic structures were performed using GenomeMatcher version 3.06 (Ohtsubo *et al.*, 2008), in which BLASTN of all-against-all bl2seq similarity searches was conducted with the parameter set ‘-F F -W 21 -e 1.0e-10’.

### Phylogenomic ana­lysis

Single-copy orthologous proteins shared among Secondary_AM, ten lineages of *Psyllophila* from four *Cacopsylla* spp. (six from *C. melanoneura*, two from *C. picta*, and one each from *C. pyricola* and *C. pyri*) (Dittmer *et al.*, 2023), 33 other insect nutritional endosymbionts belonging to the order *Enterobacterales*, and *Pseudomonas oryziphila* and two strains of *Pseudomonas entomophila* (both *Gammaproteobacteria*: *Pseudomonadales*) as outgroups were identified using OrthoFinder version 2.5.5 (Emms and Kelly, 2019). The amino acid sequences of the identified proteins were aligned with MAFFT 7.452 (Katoh and Standley, 2013) using the E-INS-i algorithm with the aid of Perl script MultiMafft.pl (https://fish-evol.org/MAFFT.html). After removing amino acid sites corresponding to alignment gaps, multiple fasta alignments were concatenated into a partitioned supermatrix using‍ ‍catsequences version 1.4 (https://github.com/ChrisCreevey/catsequences.git). Phylogenetic trees were inferred by the Maximum Likelihood (ML) method using RAxML-NG version 1.2.1 (Kozlov *et al.*, 2019) with 1,000 replicates with the MTREV+I+G4+F model, which was selected using ModelTest-NG version 0.1.7 (Darriba *et al.*, 2020). Trees were visualized using Interactive Tree of Life (iTOL) version 5 (Letunic and Bork, 2021).

### Average nucleotide identity (ANI) ana­lysis

To measure the nucleotide-level genomic similarity of *A. mori* symbionts to their counterparts in four *Cacopsylla* spp. (ten *Psyllophila* lineages as described above and eight *Carsonella* lineages from *C. melanoneura*, two from *C. picta*, and one each from *C. pyricola* and *C. pyri*), ANI was calculated using the ANI calculator (Rodriguez-R and Konstantinidis, 2016). The calculation was performed using reciprocal best hits (two-way ANI) between two genomic datasets with the default parameters. Window and step sizes were set to 1,000 and 200 bp, respectively.

### Substitution rate ana­lysis

Amino acid sequences were deduced from the protein-coding genes (CDSs) shared between *A. mori* symbionts and their counterparts in the melET isolate of *C. melanoneura*, which were then aligned with MAFFT version 7.452 (Katoh and Standley, 2013) using the E-INS-i algorithm with default parameters. The resulting protein alignments were converted to nucleotide alignments using PAL2NAL version 13.0 (Suyama *et al.*, 2006). Non-synonymous (*dN*) and synonymous (*dS*) substitution rates and *dN*/*dS* ratios between orthologous pairs were calculated using the KaKs_Calculator 1.2 package (Zhang *et al.*, 2006) with the YN model (Yang and Nielsen, 2000). All statistical ana­lyses were performed using R‍ ‍software version 4.2.1 (R Core Team 2022, https://www.r-project.org).

## Results and Discussion

### The Secondary_AM genome is as small and AT-rich as *Carsonella* genomes

**Fig. 1.**
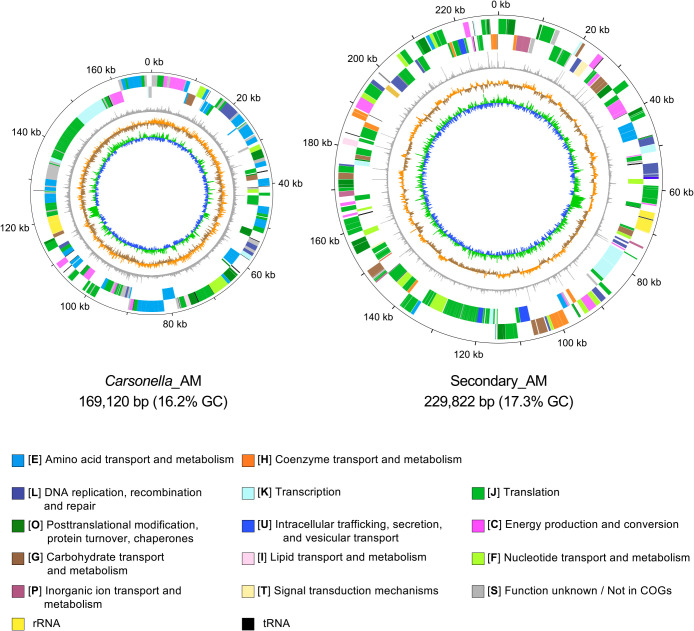
Circular representation of Secondary_AM and *Carsonella*_AM genomes. Concentric rings denote the following features (from the outside): (i) the scale in kilobases, (ii) forward strand genes, (iii) reverse strand genes, (iv) the dinucleotide bias, (v) GC skew, and (vi) G+C content. To calculate (iv), (v), and (vi), sliding windows of 100 bp and a step size of 10 bp were used.

MiSeq sequencing of *A. mori* bacteriome DNA libraries yielded 1.36 million paired-end reads (754‍ ‍Mbp) and 1.03 million mate-pair reads (447‍ ‍Mbp). We collected sequence reads that showed detectable similarity (BLASTN e-value scores <1.0E-5) to published *Carsonella* genomes, aiming to elucidate the *Carsonella*_AM genome. The assemblage of the 126,000 paired-end reads (72.6‍ ‍Mbp) and 25,000 mate pair reads (5.58‍ ‍Mbp) that were obtained and refined yielded five scaffolds and 220 large contigs (>0.5‍ ‍kbp). After filling gap sequences *in silico*, we unexpectedly succeeded in obtaining circular complete genomes with a coverage of *ca.* 100× of not only *Carsonella*_AM (169,120 bp, 16.2% GC), but also Secondary_AM (229,822 bp, 17.3% GC) (Table 1, S1, and S2, Fig. 1, S1), which were identified by the presence of known sequences of 16S rRNA genes (Fukatsu and Nikoh, 1998; Nakabachi *et al.*, 2022b). Notably, the size and GC content of the Secondary_AM genome were similar to those of *Carsonella* lineages, including *Carsonella*_AM, implying an evolutionarily ancient and obligate mutualistic relationship of Secondary_AM with the host insect. These features were similar to those of the recently published genomes of *Psyllophila* (221,413–237,114 bp, 17.3–18.6% GC), the bacteriome-associated‍ ‍sec­ondary symbiont of *Cacopsylla* spp. (Psyllidae: Psyllinae) (Dittmer *et al.*, 2023), which was in contrast to previ­ously‍ ‍sequenced markedly larger genomes with less nucleotide composition bias derived from the secondary symbionts of two psyllid species, *Ct. eucalypti* (Aphalaridae: Spondyliaspidinae) (1.4‍ ‍Mb, 43.3% GC) and *H. cubana* (Psyllidae: Ciriacreminae) (1.1‍ ‍Mb, 28.9% GC) (Sloan and Moran, 2012).

### Secondary_AM is sister to *Psyllophila*

**Fig. 2.**
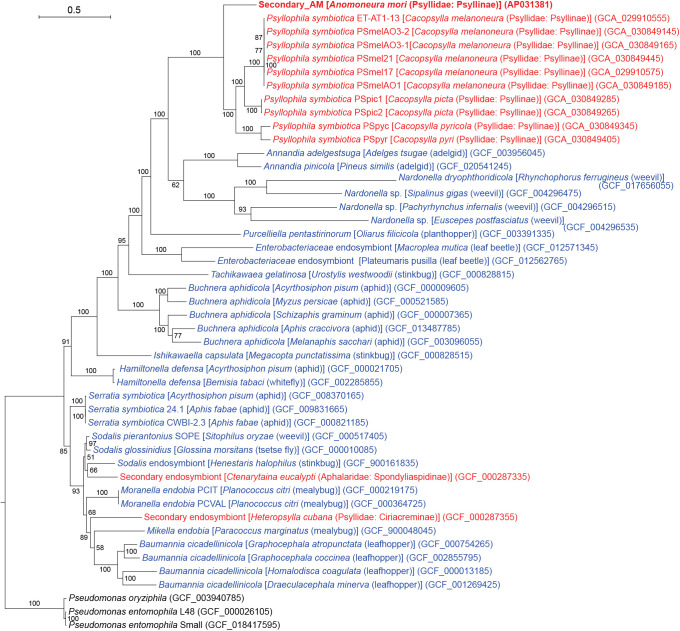
Maximum likelihood phylogram showing phylogenetic relationships among Secondary_AM, 10 *Psyllophila* lineages from four *Cacopsylla* spp., and 33 other insect endosymbionts belonging to the order Enterobacterales. For simplicity, species names are presented without “*Candidatus*.” A total of 14,766 unambiguously aligned amino acid positions derived from 60 single-copy orthologous proteins shared among these bacteria were subjected to the ana­lysis. On each branch, bootstrap support values are shown. The scale bar indicates substitutions per site. Regarding symbiotic bacteria, host organisms are shown in brackets. Symbionts of animals other than psyllids are shown in blue, while symbionts of psyllids are shown in red. The sequence from the present study is shown in bold. DDBJ/EMBL/GenBank accession numbers are provided in parentheses. *Pseudomonas oryziphila* and two strains of *Pseudomonas entomophila* (all *Gammaproteobacteria*: *Pseudomonadales*) were used as an outgroup.

Previous microbiome and phylogenetic ana­lyses using 16S rRNA genes suggested that Secondary_AM and *Psyllophila* are related and comprise a lineage widely distributed in *Cacopsylla* spp. and also in some species of other‍ ‍genera, including *Anomoneura* and* Cyamophila* (all Psyllidae: Psyllinae) (Nakabachi *et al.*, 2022b). This implies that the lineage is ancient and was acquired before the divergence of these psyllid genera. In this context, we performed a more detailed phylogenetic ana­lysis using the sequences for orthologous proteins encoded in these genomes. A total of 14,766 amino acid positions derived from 60 single-copy orthologous proteins that were found to be shared among analyzed symbionts (Table S2) were used in an ML ana­lysis (Fig. 2). The results obtained revealed that Secondary_AM formed a robustly supported clade (bootstrap: 100%) with a clade formed by 10 *Psyllophila* lineages derived from *Cacopsylla* spp., which was also robustly supported (bootstrap: 100%) in the ML tree. This branching pattern indicates that Secondary_AM is a sister lineage of *Psyllophila*, sharing a recent common ancestor. The clade of Secondary_AM and *Psyllophila* further formed a robustly supported clade (bootstrap: 100%) with “*Candidatus* Annandia” spp., the bacteriome-associated symbionts of adelgids (Hemiptera: Sternorrhyncha: Phylloxeroidea: Adelgidae), and “*Candidatus* Nardonella” spp., the bacteriome-associated symbionts of weevils (Coleoptera: Curculionoidea). The previously sequenced secondary symbionts of *Ct. eucalypti* and *H. cubana* were shown to be distantly related to this clade encompassing Secondary_AM and *Psyllophila* (Fig. 2).

ANI was calculated to further assess genomic similarities between Secondary_AM and 10 *Psyllophila* lineages (Table 2). As a comparison, ANI was also calculated between *Carsonella*_AM and 12 *Carsonella* lineages from *Cacopsylla* spp. The results obtained showed that 1) the mean ANI is higher in *Carsonella* (86.915±0.005%) (mean±standard deviation [SD]; *n*=12) than in *Psyllophila* (79.571±0.004%; *n*=10) (*P*<0.001, Welch’s *t*-test), and 2) ANI values are similar within *Carsonella* or *Psyllophila* (see SD of 1 and Table 2). However, since the ANI of Secondary_AM was the highest (79.78%) with the *Psyllophila* lineage derived from the melET isolate of *C. melanoneura* (PSmelET) (Table 2), we compared their sequences in more detail featuring this lineage.

### Secondary_AM and *Psyllophila* exhibit conserved genomic synteny

**Fig. 3.**
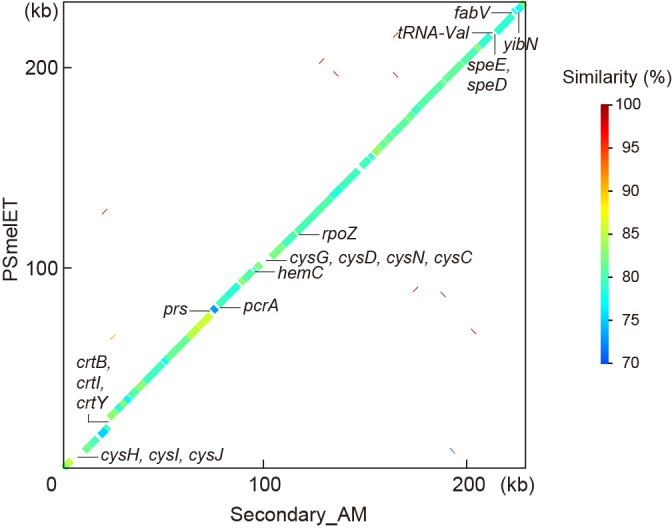
Comparison of genomic structures of Secondary_AM and *Psyllophila* derived from* Cacopsylla melanoneura* (PSmelET). The genomes of Secondary_AM and PSmelET are represented by the x and y axes, respectively. The thick line indicates shared synteny between the two genomes. The color of the line indicates percentage similarity between the nucleotide sequences. The genes found in Secondary_AM but not in PSmelET are presented below the line plot; the genes present in PSmelET but not in Secondary_AM are shown above the line plot.

Genome-wide alignments revealed highly conserved synteny between the genomes of Secondary_AM and 10 *Psyllophila* lineages (alignment with the PSmelET genome is shown as a representative in Fig. 3), indicating that most genes are shared between these symbionts and essentially no genome rearrangements have occurred since they diverged. Between the genomes of Secondary_AM and PSmelET, 230 pairs of orthologous genes were 79.8% identical at the nucleotide level and the amino acid sequences of 200 pairs of orthologous proteins were 69.0% identical on average (Table S2). They shared all genes involved in synthesizing biotin and riboflavin or complementing essential amino acid biosynthesis by *Carsonella*, which will be further discussed later.

**Fig. 4.**
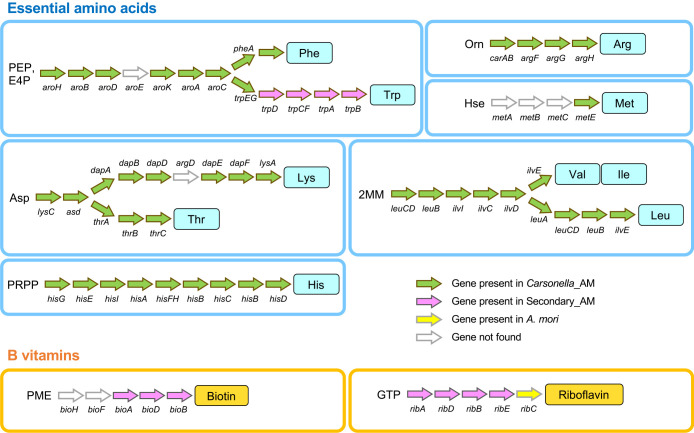
Pathways for synthesizing essential amino acids and B vitamins reconstructed from genes encoded in *Carsonella*_AM and Secondary_AM genomes. Genes denoted with green and magenta arrows are found in the genomes of *Carsonella*_AM and Secondary_AM, respectively, while those shown with white arrows appear to be absent in these genomes. *ribC* shown with a yellow arrow is suspected to be encoded in the host psyllid genome, which was horizontally acquired from an unknown bacterium. PEP, phosphoenolpyruvate; E4P, erythrose 4-phosphate; 2MM, 2-methylmalate; PRPP, phosphoribosyl diphosphate; PME, pimelyl-(acyl-carrier protein) methyl ester.

Despite this high level of conservation between the genomes, random gene silencing appeared to be ongoing in Secondary_AM and *Psyllophila*. The genes found in one of the lineages, but not in the other, are shown in Fig. 3. Neither the G+C content nor CAI of these genes was significantly different (*P*>0.05, Welch’s *t*-test) from those of genes shared between Secondary_AM and *Psyllophila* (Table S3), showing no signs of recent horizontal acquisition, which strongly suggested that the different gene inventories reflect gene silencing on either genome. *cysCDHIJN* encoding enzymes for sulfur assimilation were retained in Secondary_AM but were missing in PSmelET and all other *Psyllophila* lineages (Fig. 3 and S2, Table S2). These genes potentially contribute to the synthesis of the sulfur-containing amino acids, cysteine and methionine, the latter of which is an essential amino acid that the host insects cannot synthesize. However, no other genes related to their synthesis were retained in the Secondary_AM genome (Fig. S2 and Table S2). Moreover, *Carsonella*_AM lacked genes for the biosynthesis of cysteine/methionine other than *metE*, which converts homocysteine into methionine (Fig. 4 and S2). Therefore, the cysteine/methionine synthesis pathway appeared to be incomplete, making the role of the conservation of *cysCDHIJN* genes in Secondary_AM unclear.

On the other hand, Secondary_AM lacked genes for the synthesis of carotenoids (*crtB*, *crtI*, and *crtY*) (Fig. 3), all of which were retained in all *Psyllophila* lineages (Dittmer *et al.*, 2023). These genes were also retained in *Profftella*, a distantly related symbiont whose primary role appears to be the protection of the holobiont from natural enemies (Nakabachi *et al.*, 2013b, 2020b), presenting an example of intriguing convergence. Carotenoids are organic pigments found in diverse organisms (Mussagy *et al.*, 2019). In animals, including insects, they are supposed to play important roles, including as antioxidants and pigments for photoprotection, ornamentation, or camouflage. Although various microbes and plants produce carotenoids, metazoa cannot generally synthesize carotenoids and must acquire them through diet (Mussagy *et al.*, 2019). Since all *Carsonella* lineages sequenced to date lack genes for synthesizing carotenoids (Nakabachi *et al.*, 2006, 2013b, 2020b; Sloan and Moran, 2012; Dittmer *et al.*, 2023), the carotenoid biosynthetic genes of *Psyllophila* and *Profftella* presumably supplement host requirements. The lack of these genes in Secondary_AM may reflect the availability of carotenoids in the phloem sap of the host plant.

### *Carsonella*_AM and Secondary_AM are metabolically interdependent

Among the COG functional categories assigned to the protein-coding genes of *Carsonella*_AM and Secondary_AM, “translation” (category J) exhibited the highest percentage in both symbionts (33.2% in *Carsonella*_AM, 45.5% in Secondary_AM), while those for “transcription” (category K, 2.5% in *Carsonella*_AM, 3.6% in Secondary_AM) and “DNA replication, recombination and repair” (category L, 2.5% in *Carsonella*_AM, 6.4% in Secondary_AM) were low (Fig. S1). This result implies that translation is an especially important information-processing process for retaining the autonomy of symbionts within the host cell. In *Carsonella*_AM, the second most prominently represented COG category was E “amino acid transport and metabolism” (21.3%) (Fig. S1). Similar to other *Carsonella* strains previously analyzed (Nakabachi *et al.*, 2006, 2013b, 2020b; Nakabachi, 2008; Sloan and Moran, 2012; Dittmer *et al.*, 2023), *Carsonella*_AM retained most of the genes required for the biosynthesis of essential amino acids; however, some appeared to be missing (Fig. 4 and S2, Table S1). Regarding the tryptophan biosynthesis pathway, only *trpE* and *trpG*, genes for anthranilate synthase components involved in the first catalytic step after diverging from that for phenylalanine, were retained in the *Carsonella*_AM genome. All other genes (*trpD*, *trpCF*, *trpA*, and *trpB*) required for the remainder of the pathway were missing. Notably, these genes lost in *Carsonella*_AM were entirely retained in the Secondary_AM genome (Fig. 4 and S2). Moreover, these were the only Secondary_AM genes directly involved in the synthesis of essential amino acids, underlining the elaborate metabolic complementarity encoded in the genomes of *Carsonella*_AM and Secondary_AM. The interdependent biosynthetic pathway of tryptophan, involving *Carsonella* encoding only *trpE* and *trpG*, complemented by‍ ‍a secondary symbiont, was observed not only in *Cacopsylla* spp., but also in *Ct. eucalypti* (Aphalaridae: Spondyliaspidinae) and *H. cubana* (Psyllidae: Ciriacreminae) with distantly related and possibly more recently acquired *Enterobacterales* symbionts with larger genomes (Sloan and Moran, 2012), exemplifying other cases of convergence.

Although only 5.5% of genes encoded in the Secondary_AM genome were assigned COG category H “coenzyme transport and metabolism,” they were *bioA*, *bioD*, and *bioB*, which are required for the synthesis of the B vitamin, biotin, and *ribA*, *ribD*, *ribB*, and *ribE*, genes for the synthesis of another B vitamin, riboflavin (Fig. 4). This result suggests that supplementation of B vitamins, which are also scarce in the phloem sap diet (Ziegler *et al.*, 1975), is the pivotal role of Secondary_AM. All of these genes were retained not only in *Psyllophila*, but also in *Profftella*, further adding an example of intriguing convergence (Fig. S2). Although all the lineages of Secondary_AM, *Psyllophila*, and *Profftella* lacked *ribC*, which is required for the final step of riboflavin biosynthesis, previous screening of the genomic/transcriptomic data of several divergent psyllid lineages demonstrated that host psyllids horizontally acquired *ribC* from an uncertain bacterial lineage before the radiation of major psyllid lineages (Sloan *et al.*, 2014; Nakabachi, 2015), which is expected to complement its absence in bacterial symbionts (Fig. 4). The involvement of host genes horizontally acquired from bacteria (mostly lineages not leading to extant bacteriome residents) in metabolic pathways with symbionts has been demonstrated in the bacteriome symbioses of various hemipterans (Nakabachi *et al.*, 2005, 2014; Nikoh *et al.*, 2010; Shigenobu *et al.*, 2010; Husnik *et al.*, 2013; Luan *et al.*, 2015; Ren *et al.*, 2020; Smith *et al.*, 2021), implying its importance in the evolution of this type of intimate symbiosis. Moreover, newly acquired symbionts repeatedly add or replace the ability to synthesize B vitamins in diverse plant-sap feeding insects (McCutcheon *et al.*, 2009; Nakabachi *et al.*, 2013b, 2020b; Monnin *et al.*, 2020; Michalik *et al.*, 2021), showing that microbial supplementation with B vitamins is important, if not essential, for these insects (Nakabachi and Ishikawa, 1999).

While metabolic complementarity between *Carsonella*_AM and Secondary_AM was shown as described above, the vulnerability of these symbionts was simultaneously indicated. Regarding the COG categories for energy production and the metabolism of carbohydrates, nucleotides, and lipids (C, G, F, and I), a limited number of genes were retained, apparently constituting incomplete pathways (Fig. S1, Table S1 and S2). Furthermore, these symbionts lacked genes for many functional categories, including A, “RNA processing and modification,” M, “Cell envelope biogenesis,” D, “Cell division and chromosome partitioning,” V, “Defense mechanisms,” and Q “Secondary metabolites biosynthesis, transport and catabolism,” indicating their limited ability. The absence of Secondary_AM/Psyllophila genes involved in defense and secondary metabolism further emphasizes *Profftella*’s uniqueness in having both nutritional and defensive roles (Fig. S1, Table S1 and S2).

### Secondary_AM/*Psyllophila* genomes are more labile than *Carsonella* genomes

**Fig. 5.**
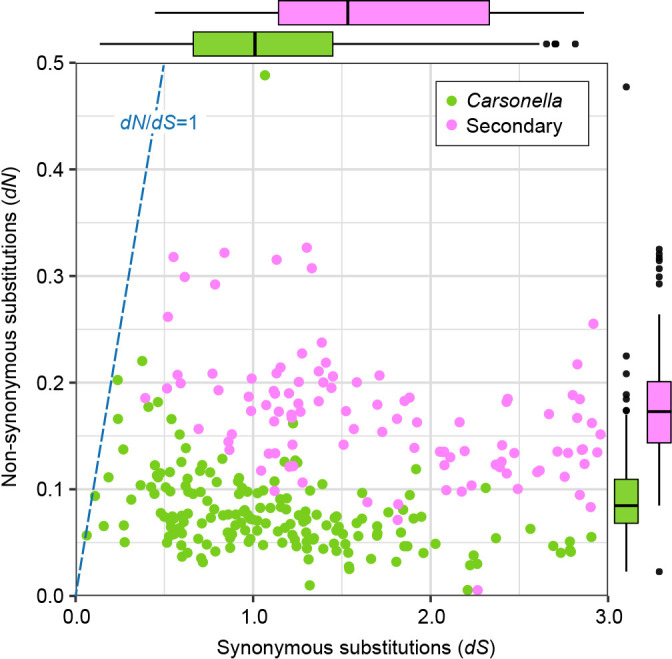
Synonymous (*dS*) and non-synonymous (*dN*) substitution rates inferred from pairwise comparisons of orthologous CDSs in Secondary_AM/*Psyllophila* lineages (magenta dots) and *Carsonella* (green dots). The values of genes with *dS*<3.0 (104 and 172 orthologous pairs in Secondary_AM/*Psyllophila* and *Carsonella*, respectively), which were used to calculate *dN*/*dS*, are shown. Box plots (Secondary_AM/*Psyllophila*, magenta; *Carsonella*, green) on the x- and y-axes indicate the distributions (median, quartiles, minimum, maximum, and outliers) of *dS* and *dN* values, respectively.

Although diverse lineages of insect primary symbionts have been shown to have highly stable genomic structures (Tamas *et al.*, 2002; Rio *et al.*, 2012; Sloan and Moran, 2012; Mao *et al.*, 2017), this is generally not the case for more recently acquired secondary symbionts (Bennett *et al.*, 2014; Campbell *et al.*, 2015; McCutcheon *et al.*, 2019; Nakabachi *et al.*, 2020b). In comparisons between symbionts of *A. mori* and their counterparts in the melET isolate of *C. melanoneura*, for which the same divergence time is assumed to be applicable, ANI (Table 2), genome-wide synteny (Fig. 3 and S3), and the similarity of orthologous genes (87.3±9.9% [mean±SD; *n*=215] for *Carsonella* vs. 79.8±9.5% for Secondary_AM/*Psyllophila* [*n*=230]) (Table S1 and S2) suggested that Secondary_AM/*Psyllophila* genomes are more labile than *Carsonella* genomes. To further assess genomic stability, we analyzed the genome-wide rates of synonymous (*dS*) and non-synonymous (*dN*) substitutions for these symbionts (Fig. 5, Table S1 and S2). Orthologous protein-coding genes, namely, 185 pairs of *Carsonella* genes and 200 pairs of Secondary_AM/*Psyllophila* genes, were used in the ana­lysis. The mean values for both *dN* (0.076±0.046 for *Carsonella* vs. 0.148±0.053 for Secondary_AM/*Psyllophila*) and *dS* (2.84±12.34 for *Carsonella* vs. 13.16±30.36 for Secondary_AM/*Psyllophila*) were higher (*P*<0.001, Brunner-Munzel test) in the Secondary_AM/*Psyllophila* lineage than in *Carsonella*. Synonymous divergence appeared to be saturated (*dS*>3.0) in 15 (8.1%) *Carsonella* genes and 95 (47.5%) Secondary_AM/*Psyllophila* genes (Table S1 and S2). To be conservative, only genes with *dS*<3.0 were used to calculate *dN*/*dS*, which was also higher (*P*<0.001, Brunner-Munzel test) in the Secondary_AM/*Psyllophila* lineages (0.134±0.111, *n*=105) than in *Carsonella* (0.121±0.151, *n*=170) (Fig. 5, Table S1 and S2). However, no gene was estimated to have *dN*/*dS*>1, indicating purifying selection for all genes analyzed in these symbionts. These results further indicate that the Secondary_AM/*Psyllophila* genomes are more labile, though still functional, than those of *Carsonella* genomes. The tendency that genomes of younger symbionts are less stable than those of ancient symbionts may lead to the preferential replacement of secondary symbionts, retaining the primary symbionts. Indeed, while the Secondary_AM/*Psyllophila* lineage appears to be widely distributed in *Cacopsylla* and other psyllid genera, several *Cacopsylla* spp. lack the lineage and harbor other secondary symbionts (Nakabachi *et al.*, 2022b), suggesting relatively recent replacements.

## Citation

Yasuda, Y., Inoue, H., Hirose, Y., and Nakabachi, A. (2024) Highly Reduced Complementary Genomes of Dual Bacterial Symbionts in the Mulberry Psyllid *Anomoneura mori*. *Microbes Environ ***39**: ME24041.

https://doi.org/10.1264/jsme2.ME24041

## Supplementary Material

Supplementary Material 1

Supplementary Material 2

Supplementary Material 3

## Figures and Tables

**Table 1.** Genomic features of bacteriome-associated symbionts in *A. mori*

	*Carsonella*_AM	Secondary_AM
Chromosome (bp)	169,120	229,822
G+C content (%)	16.2	17.3
CDS	196	215
rRNA	3	3
tRNA	28	26
Pseudogene	0	2

**Table 2.** Average nucleotide identity between symbionts of *A. mori* and *Cacopsylla* spp.

Host species	*Cacopsylla melanoneura*								*C. picta*		*C. pyricola*	*C. pyri*
Strains	melAO1	melAO2	melAO3-1	melAO3-2	melET	melST4	melST17	melST21	pic1	pic2	pyc	pyr
*Carsonella*	87.13	87.08	87.08	87.08	87.05	87.06	87.05	87.07	86.78	86.64	86.65	86.31
*Psyllophila*	79.75	—	79.53	79.75	79.78	—	79.60	79.63	79.70	79.75	79.08	79.14
