# Supplementary material for: Highly Reduced Complementary Genomes of Dual Bacterial Symbionts in the Mulberry Psyllid Anomoneura mori
Source: Microbes Environ. 2024 Sep 6;39(3):ME24041. doi: 10.1264/jsme2.ME24041 (PMC11427311; doi:10.1264/jsme2.ME24041)
Supplement: Supplementary file 1 — Supplementary Material 1 [file 39_24041_s1.pdf]

Microbes and Environments 39(3), 2024

Highly reduced complementary genomes of dual bacterial symbionts in the mulberry psyllid *Anomoneura mori*

Yuka Yasuda, Hiromitsu Inoue, Yuu Hirose, Atsushi Nakabachi

Supplemental Materials

Supplemental Tables

Table S1 (provided as a separate file). Gene list of *Carsonella*_AM.

Table S2 (provided as a separate file). Gene list of Secondary_AM.

Table S3. Sequence features of shared CDS and unique CDS in Secondary_AM and PSmelET.

	G+C% (mean±SD)	CAI (mean±SD)
Secondary_AM, shared CDS	17.3±4.5	0.91±0.03
Secondary_AM, unique CDS	18.4±3.4	0.89±0.06
PSmelET, shared CDS	19.0±4.7	0.84±0.05
PSmelET, unique CDS	18.2±5.1	0.84±0.06

Supplemental Figures

Fig. S1 (p2). COG classification of proteins encoded in *Carsonella*_AM and Secondary_AM.

Fig. S2 (p3). Amino acid and vitamin biosynthetic genes encoded in *Carsonella* and secondary symbionts from *Anomoneura mori* (AM), *Cacopsylla melanoneura* (CM), and *Diaphorina citri* (DC).

Fig. S3 (p4). Comparison of genomic structures of *Carsonella*_AM and *Carsonella* derived from *Cacopsylla melanoneura* (CRmelET).

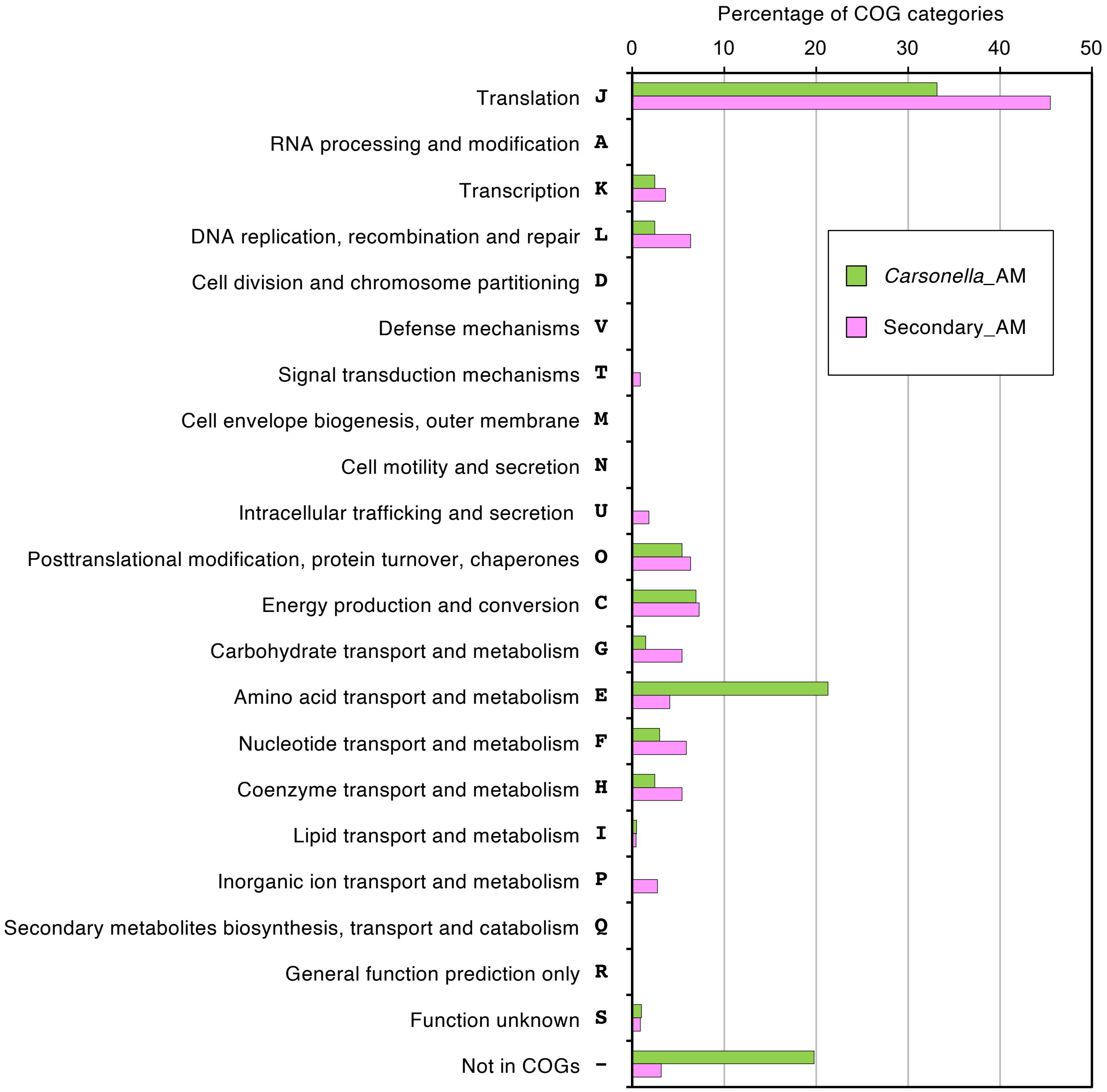

Fig. S1. COG classification of proteins encoded in *Carsonella*_AM and Secondary_AM. The percentage of the total number of genes in each functional category as defined by the COG database for *Carsonella*_AM (green) and Secondary_AM (magenta).

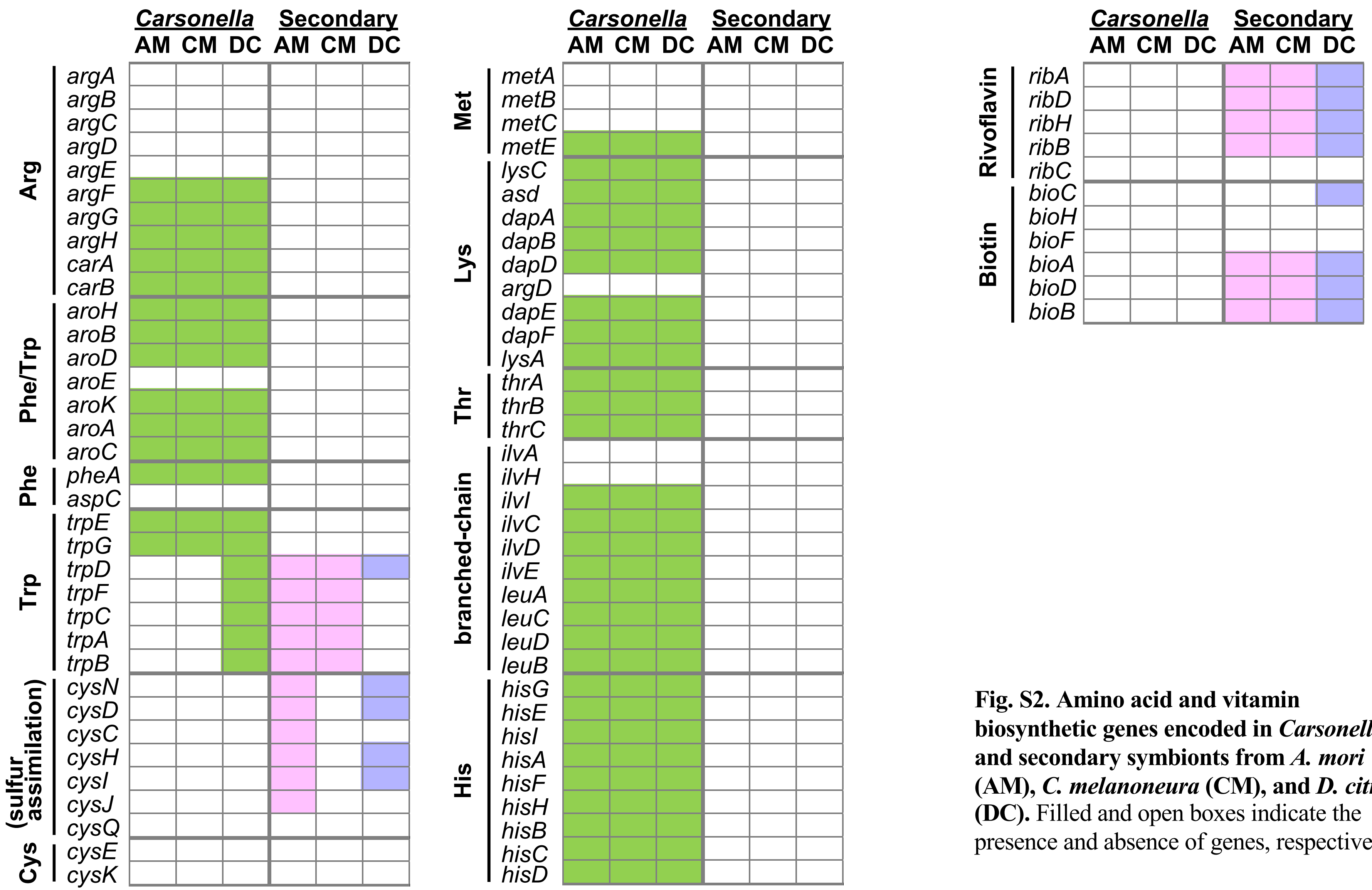

Fig. S2. Amino acid and vitamin biosynthetic genes encoded in *Carsonella* and secondary symbionts from *A. mori* (AM), *C. melanoneura* (CM), and *D. citri* (DC). Filled and open boxes indicate the presence and absence of genes, respectively.

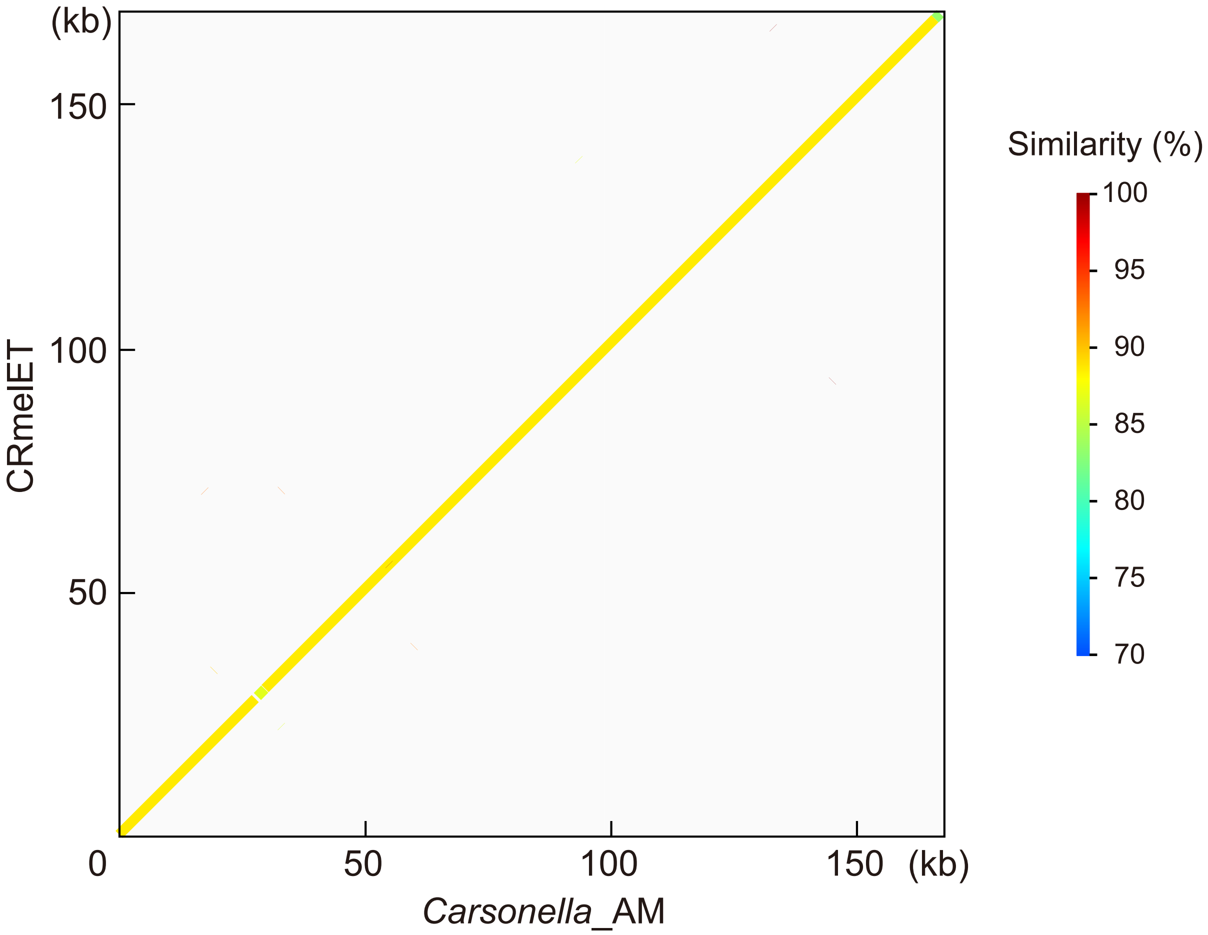

Fig. S3. Comparison of genomic structures of *Carsonella*_AM and *Carsonella* derived from *Cacopsylla melanoneura* (CRmelET). The genomes of *Carsonella*_AM and CRmelET are represented by the x and y axes, respectively. The thick line indicates the shared synteny between the two genomes. The color of the line indicates the percentage similarity between the nucleotide sequences.
